# Peripheral Nerve Injuries and Transplantation of Olfactory Ensheathing Cells for Axonal Regeneration and Remyelination: Fact or Fiction?

**DOI:** 10.3390/ijms131012911

**Published:** 2012-10-10

**Authors:** Christine Radtke, Jeffery D. Kocsis

**Affiliations:** 1 Department of Plastic, Hand- and Reconstructive Surgery, Hannover Medical School, 30625 Hannover, Germany; 2 Department of Neurology and Center for Neuroscience and Regeneration Research, Yale University School of Medicine, New Haven, CT 06510, USA; E-Mail: jeffery.kocsis@yale.edu; 3 Rehabilitation Research Center, Veterans Affairs Connecticut Healthcare System, West Haven, CT 06516, USA

**Keywords:** peripheral nerve injury, cell transplantation, olfactory ensheathing cells, axonal regeneration, remyelination, nerve defect, nerve conduit

## Abstract

Successful nerve regeneration after nerve trauma is not only important for the restoration of motor and sensory functions, but also to reduce the potential for abnormal sensory impulse generation that can occur following neuroma formation. Satisfying functional results after severe lesions are difficult to achieve and the development of interventional methods to achieve optimal functional recovery after peripheral nerve injury is of increasing clinical interest. Olfactory ensheathing cells (OECs) have been used to improve axonal regeneration and functional outcome in a number of studies in spinal cord injury models. The rationale is that the OECs may provide trophic support and a permissive environment for axonal regeneration. The experimental transplantation of OECs to support and enhance peripheral nerve regeneration is much more limited. This chapter reviews studies using OECs as an experimental cell therapy to improve peripheral nerve regeneration.

## 1. Introduction

Peripheral nerve injury results in functional deficits of peripheral targets (e.g., muscle and sensory organs) which are innervated by the injured nerve [1]. Axonal regeneration is far more successful in peripheral nerve than in the central nervous system (CNS) because inhibitory myelin proteins are less prominent in the peripheral nervous system (PNS) and Schwann cells in the distal nerve segment mobilize and establish a permissive environment for axonal regeneration. While peripheral nerve regeneration is more successful than CNS axonal regeneration, it is often incomplete; the development of interventional approaches to enhance peripheral nerve regeneration such as an adjunct cell therapy is a clinically important objective. One reason for the success of PNS regeneration is that Schwann cells are an important endogenous element in peripheral nerve regeneration and remyelination. They provide neurotrophic support and axon guidance channels for axonal regeneration and will myelinate the regenerated axons to allow rapid impulse conduction. While endogenous Schwann cells can perform these functions, additional transplantation of glia cells, such as olfactory ensheathing cells into injured nerves, may help facilitate the repair process. This may be particularly important when there is a temporal delay in repair, because the endogenous Schwann cells may atrophy and no longer appropriately signal the axon for growth thus providing less trophic support such as nerve growth factor production.

Extensive experimental OEC transplantation has been employed as a strategy to repair the injured spinal cord [2,3] and demyelinated lesions [4–7]. Furthermore, clinical studies evaluating OEC transplantation for spinal cord injury are ongoing [8–11]. However, the number of OEC studies for peripheral nerve injury is much more limited (for overview see Table 1).

OECs have been studied in the context of enhancing repair of peripheral nerve by direct transplantation in different peripheral nerve lesion models for enhancement of axonal nerve regeneration by providing a scaffold for the regenerating axons as well as trophic factors and directional cues [12]. OECs are known to provide trophic factors conducive to axonal regeneration and survival. They may promote endogenous Schwann cell mobilization possibly by a trophic influence [13,14] and can form cellular bridges in CNS white matter through which axons can regenerate [7,15]. The CNS is less permissive for axonal regeneration and sprouting than peripheral nerve. Furthermore, the introduction of OECs into the injured CNS leads a more permissive environment with reduced myelin inhibitory molecules resulting in enhanced regeneration.

Most experimental studies using OECs as a cell therapy have focused on spinal cord injury. A recent Pubmed search indicates that while there have been over 560 publications related to olfactory ensheathing cells the large majority of these studies are related to spinal cord injury. Only 27 OEC publications are related to transplantation of OECs in peripheral nerve injury models (See Table 1). Several lesion models of peripheral nerve injury have been used to study the potential of OEC transplantation to enhance nerve repair. OECs have been transplanted into sciatic nerve injury models including nerve crush [16] and nerve transection [18–20]. OECs have also been seeded on conduit implantations for nerve defect repair [21,23,36,40]. Another peripheral nerve lesion model where OECs have been transplanted is the injured facial nerve [12,26–29]. This later model has the advantage that motor recovery can be easily assayed by vibrissae movement. OECs have also been used in dorsal root injury models where the potential of sensory neurons to regenerate into the spinal cord has been studied [35,38,39]. One study used OECs for vagus nerve repair [32] and one study for ventral root repair [3]. In contrast to spinal cord repair by OEC cell transplantation, the peripheral nerve injury model studies have focused exclusively on rodents and have not as yet been transferred to larger animal models (e.g., rabbit, sheep, monkey). Moreover, while several clinical studies for spinal cord injury have been carried out, OEC clinical studies for peripheral nerve repair have not yet been initiated. In the following sections we review results from these limited studies of OECs in peripheral nerve repair.

## 2. OEC Transplantation into Sciatic Nerve Supports Axonal Regeneration and Remyelination

**Figure 1:**
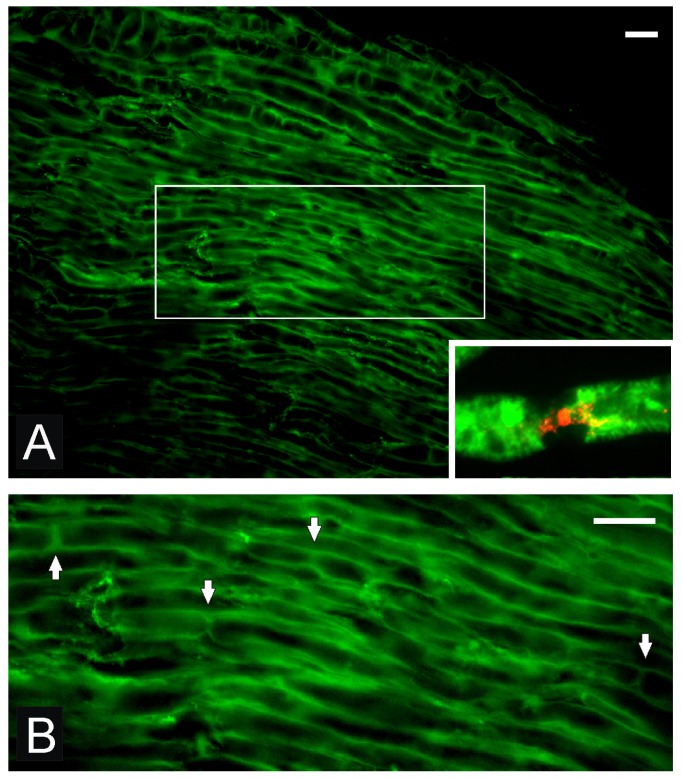
(**A**) Regenerated axons are myelinated by transplanted GFP-OECs. (**B**) Boxed image from (**A**) shows nodes of Ranvier (arrows) of the regenerated axons remyelinated by the transplanted OECs. Inset in B shows Na channel immunostaining at the newly formed node of Ranvier. Scale bar in A is 10 μm. Scale bar in B is 80 μm. (Modified with permission from Radtke *et al.* [20])

OECs prepared as cell suspension from the olfactory bulb [16,18,20] or the olfactory mucosa [19] have been transplanted directly into injured nerve. Dombrowski *et al*. [16] transplanted OECs into injured peripheral nerve (crush injury) to determine if the OECs could survive and myelinate the regenerated axons and determined additionally sodium channel expression and formation of nodes of Ranvier. Structural analysis of the regenerated axons in terms of nodal sodium channels was analyzed and results indicated that transplanted OECs integrate into peripheral nerve transected by crush injury, form peripheral-like myelin on regenerated peripheral nerve fibers and that the OECs are able to signal the regenerated axons to reconstruct nodes of Ranvier (Figure 1A,B) with proper sodium channel (Nav1.6) organization (Figure 1A, inset).

**Figure 2:**
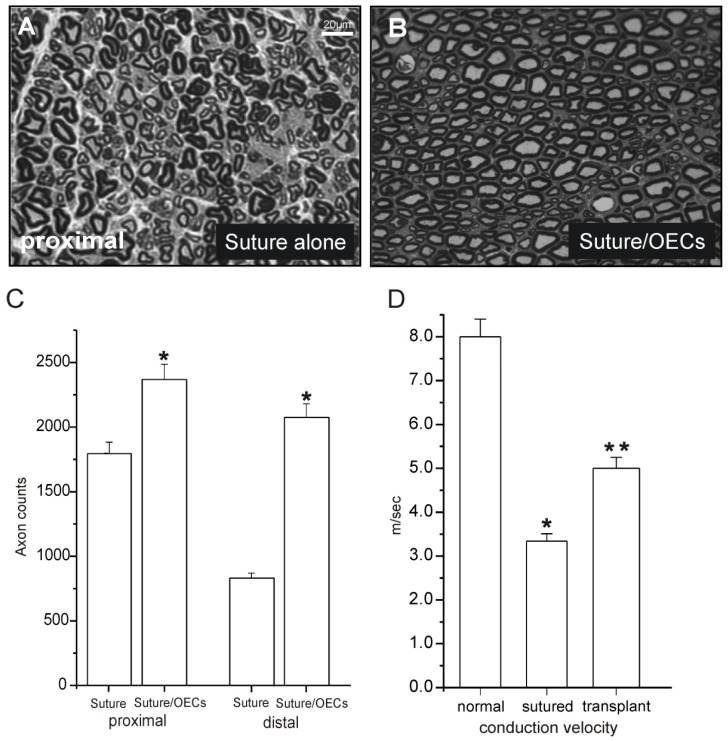
Coronal sections of repaired nerves by suture alone (**A**) and microsurgical repair combined with OEC transplantation (**B**) at three weeks post surgery. Increased numbers of myelinated axons after transplantation of OECs in the proximal segment can be observed. (**C**) and (**D**): Histological and electrophysiological outcomes between sham control (suture alone) and transplant (suture combined with OEC transplantation) animals. The number of myelinated axons (**C**) and the conduction velocity (**D**) were increased 36 days after surgery. Data are presented as means ± SE. Statistical evaluations were based on two-tailed *t*-test, χ^2^ test (Origin; criterion, * and ** *p* < 0.05). Scale bar in A = 20 μm. (Modified with permission from Radtke *et al*. [20])

In a subsequent study, combined microsurgical suture repair of the completely transected sciatic nerve with OEC transplantation was performed, and structural and functional outcomes were assessed [20]. The results of this study indicated that OEC transplantation used as an adjunct approach to microsuture repair results in improved structural (Figure 2A–C) outcome. Quantitatively measurements of myelinated axons in the OEC implanted nerves demonstrated an increase in myelinated axons after transplantation of OECs. Moreover, there was functional improvement greater than in surgical repair with vehicle injection as assayed with foot print analysis. The modest improvement in function at early (1–2 weeks) post-repair time points may be from facilitation of regeneration to more proximal musculature.

What might account for this improvement in nerve repair with combined OEC transplantation? While endogenous Schwann cells are intrinsic facilitators of peripheral nerve repair, they require several days to mobilize after nerve injury as they retract from injured or degenerating axons and subsequently express the low affinity p75 nerve growth factor receptor (p75NGFR), nerve growth factor (NGF) and other molecules which are conducive to axonal regeneration. Cultured OECs are “primed” and express these factors at the time of transplantation. After nerve transection the cut axons die-back for several millimeters over the course of several days. The axons then sprout and regenerate axons that attempt to navigate the lesion domain and reinnervate peripheral targets. The transplanted OECs may provide immediate trophic support which could account for the improved regeneration. Moreover, if there is reduced axonal die-back and earlier regeneration onset from the proximal nerve stump associated with OEC transplantation at the time of repair, the axons may be able to navigate the repair site before significant scar formation ensues. In a recent paper Guerout *et al*. [24] demonstrate significant enhancement of nerve regeneration after a severe sciatic nerve lesion and transplantation of OECs. They observed a significant increase in neurotrophic factors in the transplanted group arguing for a neurotrophic effect by the transplanted cells as a facilitator of nerve regeneration. OEC transplantation can also facilitate recurrent laryngeal nerve regeneration [30,31]. One study used SCs and OECs in a nerve conduit model and found that SCs were more effective in promoting axonal regeneration [25]. Navarro *et al*. [33] found that OECs promoted dorsal root regeneration, but Ramer *et al*. [37] found that they did not.

## 3. Implantation of OEC-Seeded Scaffolds for Nerve Substance Defect Repair

The clinical outcome in long distance nerve defects is particularly disappointing and the development of interventional approaches to improve functional recovery is continuing. A complicating factor is the trauma-associated loss of nerve tissue (substance defect) where autologous nerve grafts are required, but are limited in availability. A promising alternative to conventional autologous nerve grafting as described above is the utilization of artificial nerve grafts in the form of scaffolds or conduits [21–23,41]. While the functional outcome is often suboptimal, efforts are being made to overcome these restrictions. The addition of supportive cells to the nerve tube to optimize results is an extensively investigated modification to a single-lumen nerve tube. In the repair of small nerve defects with insertion of empty hollow nerve tubes, Schwann cells are also involved in the process of regeneration by endogenous migration. The addition of OECs might further enhance regeneration. OECs were evaluated regarding their properties after seeding into a variety of scaffolds. Tang *et al*. [40] evaluated the compatibility of a collagen-heparan sulfate (CHS) biological nerve tube filled with OECs. The scaffolds were co-cultured with OECs *in vitro*. The attachment and growth of OECs in CHS scaffolds were observed indicating that the scaffold is a possible cell carrier for the implantation of OECs in nerve tissue bioengineering. Moreover, purified olfactory mucosa-derived OECs were seeded onto a bioengineered hybrid scaffold consisting of various extracellular matrix (ECM) proteins and cultured. A stable porous 3-D network was formed, and OECs seeded on the scaffold maintained the expression of nerve growth factor, matrix metalloproteinase-3 and matrix metalloproteinase-9 was studied *in vitro* [41]. In silk fiber scaffolds with different fiber diameters and seeded with OECs, characteristics of OECs were observed by analyzing cell morphological feature, distribution, and proliferation. OECs specific cell markers could be maintained and the migration including tracks, turning behavior, migration distances, migration speeds, and forward migration indices were calculated [42]. Additionally, the scaffold material itself has a noticeable effect on OEC growth and proliferation. In comparison of the copolymers PDLLA (poly-DL-lactide) and PLGL or poly[LA-co-(Glc-alt-Lys)], PLGL possesses better hydrophilicity and biocompatibility and provided a better cell growth for neonatal OECs [43].

A recent study evaluated the compatibility between the copolymer PLGA or poly (lactic-co-glycolic acid) and OECs *in vitro*, and the effect of a PLGA conduit filled with OECs and silicon-extracellular matrix gel on a 10 mm-defect in the sciatic nerve in rat [22]. The nerve conduction velocity and the amplitude of compound muscle action potential were more improved in the PLGA-guided group than in the control silicon-guided group. The PLGA-OEC conduits also had a greater number of regenerated axons. However, there was no difference between the groups in the functional outcome measured by sciatic functional index at 12 weeks after surgery, which the authors attribute to the severity of the nerve injury model [22].

In another study OECs suspended in laminin gel and seeded in a silicone tube were used to bridge a 15 mm gap in rat sciatic nerve [21]. The OEC seeded tubes were much more successful in promoting nerve regeneration than were the tubes alone. The use of nerve conduit implantation for the treatment of nerve substance defects is the subject of intensive ongoing research. Establishment of the proper combination of conduit material and cell seeding will be important to advance success for peripheral nerve tissue engineering.

### 3.1. OECs for Facial Nerve Repair

Comparable to the sciatic nerve lesion model, OECs prepared from the olfactory bulb and the olfactory mucosa were used in facial nerve lesions in rats. In these lesion models either the facial nerve was directly anastomosed or a repair with a 5 mm interstump distance combined with a silicone tube was performed [26,27]. In both studies increased sprouting and pathfinding could be observed, but no improvement of accuracy of reinnervation or functional alterations could be shown. OEC transplantation into transected facial nerve enhances axonal sprouting [12,26], promotes recovery of vibrissae motor performance [27] and increases the rate of eye closure [37]. OECs were tested in several studies for facial nerve repair. Moreover, Angelov *et al*. [28] demonstrated moderate nerve regeneration, but only olfactory mucosa resulted in functional improvement. Thus, reports of achievement of functional repair in the sciatic nerve model system with OEC transplantation have shown more success than in facial nerve repair. A recent study carried out complete rat vagus nerve lesion followed by surgical anastomosis combined olfactory bulb (OB) or olfactory mucosa (OM) OECs transplantation. Here, improvement of reinnervation was observed by EMG testing, and demonstration of increased numbers of regenerated myelinated fibers and functional improvement [32].

### 3.2. OECs in Dorsal Root Injury

Axon growth-promoting properties of OECs were determined by several studies using dorsal and ventral root lesion models in the adult rat. The lesion models include dorsal and ventral root avulsion followed by root reimplantation and as well acute and chronic transection models. However, whereas early *in vivo* studies reported facilitated entry of peripheral sensory dorsal root ganglionic axons by transplantation of OECs [2,33] other studies could not support these observations [35,37]. Additionally, Li *et al.* [44] reported beneficial effects of transplanted OECs to the reanastomosed ventral S1 root with increased fibers crossing the lesion side when OECs combined with fibroblasts were transplanted at the spinal cord-root interface. Ibrahim *et al.* [38] reported on transplantation of OECs in a brachial plexus injury model. Here, OECs increased regeneration at both the anatomical and functional level.

## 4. Concluding Remarks

Peripheral nerve injury constitutes a critical and common clinical problem. While simple nerve repairs can often lead to considerable functional improvement, clinical outcomes are not fully optimal. Experimental studies performed in rodents show that transplantation of OECs into injured nerve or implantation of OEC-seeded conduits leads to an enhancement in axonal regeneration and improved functional outcome under some experimental conditions. Axonal die-back of the proximal nerve stump is reduced in the OEC transplanted nerves suggesting that the OECs provided early trophic support leading to earlier onset of regeneration. This could be critical for allowing the regenerating axons to navigate across the injury site before impeding scar tissue develops. However, OECs share many properties with Schwann cells such as their production of neurotrophic factors and extracellular matrix molecules as well as their ability to form peripheral myelin. There are few direct comparisons between the nerve repair potential of OECs and Schwann cells. Moreover, OECs could in principle promote Schwann cell proliferation, thus having an indirect effect on nerve repair. Transplanted identified eGFP-expressing OECs integrate into the nerve injury site and remyelinate the regenerated axons, suggesting direct participation of OECs in the repair process. Yet, transplantation of Schwann cells shows similar integration emphasizing the need for studies to compare the relative repair potential of OECs and Schwann cells. Future work with biosynthetic constructs seeded with cells such as OECs will represent an important area of research for potentially establishing novel therapeutic approaches for nerve injury. Another issue with regard to comparing various studies using OECs for nerve repair, is that many of the studies use OECs prepared from different age animals (neonate *vs.* adult), from different sites of derivation (e.g., nasal muscosa *vs.* olfactory bulb) and methods of cell purification. In spite of these differences, to date the enhancement of axonal regeneration and remyelination following OEC transplantation into the injured peripheral nervous appears to be fact. Yet, many questions remain to be addressed as to the best source of OECs and the optimal culture conditions to be used prior to transplantation.

## Supplementary Materials



## Figures and Tables

**Table 1:** Summary of olfactory ensheathing cell (OEC) transplantation studies into peripheral nerve injury models.

Nerve lesion model	OEC condition	OEC application	Outcome	Limits	Reference
Sciatic nerve crush lesion (rat)	GFP-OECs purified 30,000 cells/μL and 10 μL used	OEC injection proximal and distal to lesion	myelin formation and axonal regeneration high density of Na(v)1.6 newly formed nodes of Ranvier	no functional testing performed	Dombrowski *et al*., 2006 [16]
Sciatic nerve transection and silicone entubulation (rat)	OB OECs	OECs injected in silicone tube	improvement of CMAP increased nerve fiber regeneration and thickness of myelination	no limits or side effects reported	Cheng *et al*., 2003 [17]
Sciatic nerve transaction (rat)	OB OECs	OEC injection in lesion side	enhancement of axonal regeneration reduction of motoneuron apoptosis	no significant difference in neuronal survival in experimental and control groups	Wang *et al*., 2005 [18]
Sciatic nerve transaction (rat)	olfactory mucosa transplantation	olfactory mucosa transplantation	SFI increased	Control group only nontransected animals	Delaviz *et al.*. 2008 [19]
Sciatic nerve transaction and microsurgical repair by suture (rat)	GFP-OECs purified/PKH labeled 30,000 cells/μL and 10 μL used	OECs injection proximal and distal to lesion	Axonal regeneration and remyelination newly formed nodes of Ranvier functional improvement	Observation interval limited to 3 weeks	Radtke *et al*., 2009 [20]
Sciatic nerve lesion 12–15mm gap and tube implantation (rat)	Purified PKH-labelled OB OECs 120,000 cells/tube	Silicone tubel prefilled with OECs in laminin gel	Enhancement axonal regeneration increased CMAP functional improvement	Regeneration limit at 15 mm Regeneration in 50% of animals	Verdu *et al*., 1999 [21]
Sciatic nerve lesion 10 mm PLGA conduit implantation (rat)	CM-Dil labeled OECs in 1 × 10,000 μL and 50 μL used	PLGA filled with OECs OECs in EMC	Nerve fiber regenation motor function recovery NCV and CMAP recovery	No recovery SFI after 12 weeks	Li *et al*., 2010 [22]
Sciatic nerve lesion 20 mm and PLGA conduit implantation (rat)	Purified OECs Hoechst-labelled 3 × 100,000 μL and 20 μL used	PLGA filled with OECs OECs in EMC	Enhancement axonal regeneration increased myelinated fibers recovery sensory and motor function	20% of rats showed autophagia and heel ulcers	You *et al*., 2010 [23]
Sciatic nerve lesion and 20 mm resection, no surgical repair (rat)	Cultured OECs from olfactory bulb GFP-labelled cells, purity was determined by p75NGFR	Cell suspension was laid into transaction site immediately after resection	Muscle strength and morphometric axon counting with complete restoration, increase of neurotrophic factors	OECs did not directly on axonal regrowth, but seem to create favorable microenviroment	Guerout *et al*., 2011a [24]
Sciatic nerve lesion 15 mm and biogenic conduit implantation (rat)	Purified neonatal OECs or purified neonatal SCs	Conduits filled with OECs or SCs	Improvement in motor function	Recovery better after SCs compared to OECs with conduit implantation nerve transplant best results	Penna *et al*., 2012 [25]
Facial nerve lesion (rats) 5 mm interstump distance silicone tube	OB OECs deplated of fibroblasts 200,000 OECs	Collagen gel containing OECs in silicone tube	Increased motoneurons 10 fold increase in motoneurons increased sproutuing and pathfinding	No functional alterations	Guntinas-Lichius *et al*., 2001 [26]
Facial nerve lesion (rat) end-to end anastomosis	OM freshly prepared detection by y-chromsome	OM laid over sutured epineurium	Reduction of collateral branching promatio of functional recovery sustained expression trophic factors	No improvement of accuracy of reinnervation	Guntinas-Lichius *et al*., 2002 [27]
Facial nerve lesion (rat)	OB OECs and OM transplantation	OM pieces were applied OEC suspension injected	Moderate nerve regeneration	Only OM yielded in major improvement	Angelov *et al*., 2005 [28]
Fiacial nerve lesion (rat) and immediate repair by suture	Mixed OECs and S-type OECs	Bolus of cultured cells was applied to the cut ends before suture	Increased rate of eye closure recovery	Disorganization of the facial nucleus and aberrant nerve branching unchanged	Choi and Raisman, 2005 [29]
recurrent laryngeal nerve section/anastomosis (rat)	OECs from mucosa (OM-OECs), or olfactory bulb (OB-OECs) or co-transplantation of both	Cells were laid over section/anastomosis site immediately at the time of surgery (6 ×10,000 cells)	Co-transplantation of OM-OECs and OB-OECs supported major functional recovery with reduction of synkinesis	OM-OECs or OB-OECs displayed opposite abilities to improve functional recovery	Guerout *et al*., 2011b [30]
Vagus nerve transaction and immediate repair by suture	Cultivated olfactory bulb or cultivated olfactory mucosa of non-cultivated olfact. mucosa		best vocal fold angular movement with cultivated olfactory mucosa in all cell groups less synkinesis		de Corgnol *et al*., 2011 [31]
Complete vagus nerve lesion and anastomosis in rat	GPF OM and OB OECs 5 × 1,000,000 cells/animal	OB or OM OECs in matrigel per micropipette in anatomosis side	Improvement of reinnervation (EMG) increased myelinated fibers functional improvement	OM OECs improves muscular activity but no increases in number of myelinated fibers	Pavoit *et al*., 2011 [32]
Transection of dorsal roots L3-L6 in rats	OECs from olfactory nerve and glomerular layer, immunopurified marked with PKH28	Impantation into DREZ	promotion of central regeneration and functional reconnection of regenerating sensory afferents, reflex recovery	immunoreactive fibers entering DH with lower density than contalateral side	Navarro *et al*., 1999 [33]
Dorsal root rhizotomy at C3-T3 in rats	purified OB-OECs	direct OEC transplantation dorsal horn OEC transplants or into the DREZ	axons regenerated at the rhizotomy site	no regeneration across DREZ no regeneration into dorsal horn	Gomez *et al*., 2003 [34]
Doral root entry zone/dorsal horn rhizotomy in rats	purified by p75NGFR OECs identification by β-gal 30,000–200,000 cells	injection of OEC suspension at DREZ/DH	no advantage in promoting ingrowth of afferent fibers in DREZ	no evidence of functional recovery of afferent fibers, minimal ingrowth of afferent fibers in SpC	Riddell *et al*., 2004 [35]
Dorsal root transection at L4 in rats	endogenous matrix containing GFP-OECs	direct application to surfaces of rootlet and SpC combined with fibrin glue	regenerated dorsal root axons crossed repaired DREZ	transplanted cells did not enter the spinal cord itself	Li *et al*., 2004 [36]
Cervical or lumbar dorsal root lesion in rats	GFP-OECs from lamina propria	OECs transplanted into DRG, intact or injured dorsal roots or the dorsal columns via DREZ	OECs migration into the DRG/dorsal root	OECs migrated within the PNS but did not cross the DREZ no primary afferent regeneration	Ramer *et al*., 2004 [37]
Dorsal roots transection C5-T2 acute and chronic lesion (rats)	GFP-OECs from OB	OECs injection in roots C4-T1	restoration fore-paw function recovery sensory input axonal regeneration	none of chronically rhizotomized rats showed electrophysiological responses	Ibrahim *et al*., 2009 [38]
Dorsal root injury at C7 and C8 in rats	GFP-cultures enriched for OECs 6 × 10,000 cells	stereotactic injection into dorsal horn	attenuation of neuropathic pain	no improvement sensory function increasement of selfmutilation no functional improvement	Wu *et al*., 2010 [39]
Avulsion of ventral root at S1 and reimplantation (rat)	GFP-OECs and fibroblasts 1:1	OECs transplanted at SpC interface OECs matrix cut into pieces	increase of fibers crossing lesion side migration of OECs	20% of fibers enter roots without OEC transplantation	Li *et al*., 2007 [17]

## Abbreviations

CHS: collagen-heparan sulphate
CNS: central nervous system
DREZ/DH: dorsal root entry zone/dorsal horn
ECM: extracellular matrix
EMG: electromyography
GFP: green fluorescent protein
CMAP: compound muscle action potential
Nav: voltage-gated TTX-sensitive sodium channels
Nav1.6: voltage gated sodium channel subtype 1.6
NGF: nerve growth factor
NCV: nerve conduction velocity; nerve growth factor
OB: olfactory bulb
OECs: olfactory ensheathing cells
OM: olfactory mucosa
PDLLA: poly D, L-lactic acid
PGA: polymer polyglycolic acid
PHB: poly-3-hydroxybutyrate
PLGL: poly [LA-co-(Glc-alt-Lys)]
PLLA: poly L-lactic acid
p75NGFR: p75 nerve growth factor receptor
PNS: peripheral nervous system
SFI: sciatic functional index
SpC: spinal cord
