# Supplementary material for: Peripheral Nerve Injuries and Transplantation of Olfactory Ensheathing Cells for Axonal Regeneration and Remyelination: Fact or Fiction?
Source: Int J Mol Sci. 2012 Oct 10;13(10):12911–24. doi: 10.3390/ijms131012911 (PMC3497303; doi:10.3390/ijms131012911)
Supplement: Supplementary file 1 [file ijms-13-12911-s001.pdf]

Nerve lesion model	OEC condition	OEC application	Outcome	Limits	Reference
Sciatic nerve crush lesion (rat)	GFP-OECs purified 30 000 cells/µl and 10 µl used	OEC injection proximal and distal to lesion	myelin formation and axonal regeneration high density of Na(v)1.6 newly formed nodes of Ranvier	no functional testing performed	Dombrowski et al., 2006
Sciatic nerve transection and silicone entubulation (rat)	OB OECs	OECs injected in silicone tube	improvement of CMAP increased nerve fiber regeneration and thickness of meylination	no limits or side effects reported	Cheng et al., 2003
Sciatic nerve transection (rat)	OB OECs	OEC injection in lesion side	enhancement of axonal regeneration reduction of motoneuron apoptosis	no significant difference in neuronal survival in experimental and control groups	Wang et al., 2005
Sciatic nerve transection (rat)	olfactory mucosa transplantation	olfactory mucosa transplantation	SFI increased	control group only nontransected animals	Delaviz et al., 2008
Sciatic nerve transection and microsurgical repair by suture (rat)	GFP-OECs purified/PKH labelled 30 000 cells/µl and 10 µl used	OECs injection proximal and distal to lesion	axonal regeneration and remyelination newly formed nodes of Ranvier functional improvement directional growth of axons	observation intervall limited to 3 weeks	Radtke et al., 2010
Sciatic nerve lesion 12-15mm gap and tube implantation (rat)	purified PKH-labelled OB OECs 120000 cells/tube	silicone tube prefilled with OECs in laminin gel	enhancement axonal regeneration increased CMAP functional improvement	regeneration limit at 15mm regeneration in 50% of animals	Verdu et al, 1999
Sciatic nerve lesion 10 mm PLGA conduit implantation (rat)	CM-Dil labelled OECs in 1x 10000 /µl and 50 µl used	PLGA filled with OECs OECs in EMC	nerve fiber regeneration motor function recovery NCV and CMAP recovery	no recovery SFI after 12 weeks	Li et al., 2010
Sciatic nerve lesion 20mm and PLGA conduit implantation (rat)	purified OECs Hoechst-labelled 3x 100000/µl iand 20µl used	PLGA filled with OECs OECs in EMC	enhancement axonal regeneration increased myelinated fibers recovery sensory and motor function	20% of rats showed autophagia and heel ulcers	You et al., 2010
Sciatic nerve lesion and 20mm resection, no surgical repair (rat)	cultured OECs from olfactory bulb GFP-labelled cells, purity was determined by p75NGFR	cell suspension was laid into transection site immediately after resection	muscle strength and morphometric axon counting with complete restoration, increase of neurotrophic factors	OECs did not act directly on axonal regrowth, but seem to create favorable microenvironment	Guérout et al., 2011a
Sciatic nerve lesion 15mm and biogenic conduit implatation (rat)	purified neonatal OECs or purified neonatal SCs	conduits filled with OECs or SCs	improvement in motor function	recovery better after SCs compared to OECs with conduit implantation nerve transplant best results	Penna et al., 2012
Facial nerve lesion (rats) 5mm interstump distance silicone tube	OB OECs deplated of fibroblasts 200 000 OECs	collagen gel containing OECs in silicone tube	increased motoneurons 10fold increase in motoneurons increased sproutuing and pathfinding	no functional alterations	Guntinas-Lichius et al. 2001
Facial nerve lesion (rat) end-to end anastomosis	OM freshly prepared detection by y-chromsome	OM laid over sutured epineurium	reduction of collateral branching promatio of functional recovery sustained expression trophic factors	no improvement of accuracy of reinnervation	Guntinas-Lichius et al., 2002
Facial nerve lesion (rat)	OB OECs and OM transplantation	OM pieces were applied OEC suspension injected	moderate nerve regeneration	only OM yielded in major improvement	Angelov et al., 2005
Facial nerve lesion (rat) and immediate repair by suture	mixed OECs and S-type OECs	bolus of cultured cells was applied to the cut ends before suture	increased rate of eye closure recovery	disorganization of the facial nucleus and abberant nerve branching unchanged	Choi and Raisman, 2005
recurrent laryngeal nerve section/ anastomosis (rat)	OECs from mucosa (OM-OECs), or olfactory bulb (OB-OECs) or co-transplantation of both	cells were laid over section/ anastomosis site immediately at the time of surgery (6x10.000 cells)	co-transplantation of OM-OECs and OB-OECs supported major functional recovery with reduction of synkinesis	OM-OECs or OB-OECs displayed opposite abilities to improve functional recovery	Guérout et al., 2011b
vagus nerve transection and immediate repair by suture	cultivated olfactory bulb or cultivated olfactory mucosa of non-cultivated olfact. mucosa		best vocal fold angular movement with cultivated olfactory mucosa in all cell groups less synkinesis		de Corgnol et al., 2011

Complete vagus nerve lesion and anastomosis in rat	GPF OM and OB OECs 5x1000 000 cells/animal	OB or OM OECs in matrigel per micropipette in anatomosis side	improvement of reinnervation (EMG) increased myelinated fibers functional improvement	OM OECs improves muscular activity but no increas in number of myelinated fibers	Pavoit et al., 2011
Transection of dorsal roots L3-L6 in rats	OECs from olfactory nerve and glomerular layer, immunopurified marked with PKH26	Implantation into DREZ	promotion of central regeneration and functional reconnection of regenerating sensory afferents, reflex recovery	immunoreactive fibers entering DH with lower density than contalateral side	Navarro et al., 1999
Dorsal root rhizotomy at C3-T3 in rats	purified OB-OECs	direct OEC transplantation dorsal horn OEC transplants or into the DREZ	axons regenerated at the rhizotomy site	no regeneration across DREZ no regeneration into dorsal horn	Gomez et al., 2003
Doral root entry zone/ dorsal horn rhizotomy in rats	purified by p75NGFR OECs identification by ß-gal 30 000-200 000 cells	injection of OEC suspension at DREZ/ DH	no advantage in promoting ingrowth of afferent fibers in DREZ	no evidence of functional recovery of afferent fibers, minimal ingrowth of afferent fibers in SpC	Riddell et al., 2004
Dorsal root transection at L4 in rats	endogenous matrix containing GFP-OECs	direct application to surfaces of rootlet and SpC combined with fibrin glue	regenerated dorsal root axons crossed repaired DREZ	transplanted cells did not enter the spinal cord itself	Li et al., 2004
Cervical or lumbar dorsal root lesion in rats	GFP-OECs from lamina propria	OECs transplanted into DRG, intact or injured dorsal roots or the dorsal columns via DREZ	OECs migration into the DRG/ dorsal root	OECs migrated within the PNS but did nor cross the DREZ no primary afferent regeneration	Ramer et al., 2004
Dorsal roots transection C5-T2 acute and chronic lesion (rats)	GFP-OECs from OB	OECs injection in roots C4-T1	restoration fore-paw function recovery sensory input axonal regeneration	none of chronically rhizotomized rats showed electrophysiological responses	Ibrahim et al., 2009
Dorsal root injury at C7 and C8 in rats	GFP-cultures enriched for OECs 6x10000 cells	stereotactic injection into dorsal horn	attenuation of neuropathic pain	no improvement sensory function increasement of selfmutilation no functional improvement	Wu et al., 2010
Avulsion of ventral root at S1and reimplantation (rat)	GFP-OECs and fibroblasts 1:1	OECs transplanted at SpC interface OECs matrix cut into pieces	increase of fibers crossing lesion side migration of OECs	20% of fibers enter roots without OEC transplantation	Li et al., 2007

Table 1: Summary of OEC transplantation studies into peripheral nerve injury models

sodium channel 1.6; NCV nerve conduction velocity; OB olfactory bulb; OECs olfactory ensheathing cells; OM olfactory mucosa; p75NGFR p75 nerve growth factor receptor; PLGA polylactide-co-glycolide acid;

SCs schwann cells; SFI sciatic functional index; SpC spinal cord
